# Automatic Construction of a Depression-Domain Lexicon Based on Microblogs: Text Mining Study

**DOI:** 10.2196/17650

**Published:** 2020-06-23

**Authors:** Genghao Li, Bing Li, Langlin Huang, Sibing Hou

**Affiliations:** 1 School of Information Technology & Management University of International Business and Economics Beijing China; 2 Graduate School of Art and Science Columbia University New York, NY United States

**Keywords:** depression detection, depression diagnosis, social media, automatic construction, domain-specific lexicon, depression lexicon, label propagation

## Abstract

**Background:**

According to a World Health Organization report in 2017, there was almost one patient with depression among every 20 people in China. However, the diagnosis of depression is usually difficult in terms of clinical detection owing to slow observation, high cost, and patient resistance. Meanwhile, with the rapid emergence of social networking sites, people tend to share their daily life and disclose inner feelings online frequently, making it possible to effectively identify mental conditions using the rich text information. There are many achievements regarding an English web-based corpus, but for research in China so far, the extraction of language features from web-related depression signals is still in a relatively primary stage.

**Objective:**

The purpose of this study was to propose an effective approach for constructing a depression-domain lexicon. This lexicon will contain language features that could help identify social media users who potentially have depression. Our study also compared the performance of detection with and without our lexicon.

**Methods:**

We autoconstructed a depression-domain lexicon using Word2Vec, a semantic relationship graph, and the label propagation algorithm. These two methods combined performed well in a specific corpus during construction. The lexicon was obtained based on 111,052 Weibo microblogs from 1868 users who were depressed or nondepressed. During depression detection, we considered six features, and we used five classification methods to test the detection performance.

**Results:**

The experiment results showed that in terms of the F1 value, our autoconstruction method performed 1% to 6% better than baseline approaches and was more effective and steadier. When applied to detection models like logistic regression and support vector machine, our lexicon helped the models outperform by 2% to 9% and was able to improve the final accuracy of potential depression detection.

**Conclusions:**

Our depression-domain lexicon was proven to be a meaningful input for classification algorithms, providing linguistic insights on the depressive status of test subjects. We believe that this lexicon will enhance early depression detection in people on social media. Future work will need to be carried out on a larger corpus and with more complex methods.

## Introduction

### Background

Depression, one of the major reasons for suicide in recent years, is a severe mental disorder characterized by persisting low mood states in the affected person. It is expected to be the largest contributor to disease burden worldwide by 2030, especially in China with a high-pressure lifestyle. According to a World Health Organization (WHO) report in 2017 [1], China had more than 54 million people with depression, which means that there was almost one patient with depression among every 20 people. In addition, a national estimation based on China’s 2012 census data shows that with an adult population size of 1.04 billion, an estimated 258.41 million adults (24.79%) are at increased risk of depressive symptoms [2]. It has been reported that the suicide rate among patients with depression is more than 20 times that of the general population, and patients with depression account for more than half of those who have committed suicide [3].

Diagnosis of potential depression in an early stage can provide more opportunities for those affected to receive appropriate treatment and overcome the disease. However, owing to the lack of mental health knowledge, the lack of regular counseling, and the fact that mental health diseases are greatly different from physical diseases as there is no pain, many patients with depression do not recognize it. Although some know a little about depression, they are often reluctant to seek professional help because of a sense of shame [4].

The traditional clinical diagnosis of depression mainly relies on standardized assessments, which are highly accurate but have limitations in detection efficiency [5]. The medical diagnosis requires not only filling in a depression assessment scale, such as the Self-rating Depression Scale, but also a one-to-one interview and long-term observation [6], which involve high costs. Patients tend to remain undetected until the disease presents obvious symptoms, which also means that the optimal treatment period has passed [7]. The whole diagnosis process is highly passive, as doctors have to wait for patients to knock on their door.

Things are changing with the development of social media. Nowadays, many methods combining machine learning algorithms and text mining techniques have been developed to diagnose potential depression in an early stage [8-13]. Compared with traditional approaches, these methods have been proven to be effective and inexpensive, and have been shown to reduce limitations and assist in clinical diagnosis in a more flexible way. At the same time, people are used to disclosing their inner feelings on social media. The huge corpus provides abundant text describing things like sadness, exhaustion, and breakdown, which have the potential to reflect depression. Hundreds of millions of people in third or fourth tier cities and poor mountainous areas in China have little chance to disclose their mental conditions directly to experts, but they can provide their accounts and apply for social media methods. Experts can then intervene and conduct more targeted treatments for users who are potentially depressed. Another scenario involves teenagers on campus, and teachers can pay more attention to the actual mental status of students who are potentially depressed with the help of forums and other web-based text. It is thus feasible to detect users’ depressive mental states on a large scale on social media, and this provides convenience for expert assessment.

Actually, when coping with textual depression data, word-based features like frequency and embedding are commonly used and a domain lexicon might be valuable to understand the author of the text [14]. Many research studies have achieved a lot in terms of a depression lexicon, which is mainly in English [9,12,13]. In China, research about web-related depression detection is just getting started, and we did not find any domain lexicon research about depression in a public study. It would not be proper to translate an English lexicon directly owing to cultural differences. Thus, a depression-domain lexicon in Chinese is needed.

In this paper, based on a well-labeled depression data set on Weibo, which is one of the largest Chinese user-generated content platforms, we constructed a depression-domain lexicon containing more than 2000 words. This lexicon can be used to assist in the early diagnosis of depression. We crawled more than 144,000 microblog tweets of nearly 2000 users within a time span of 16 months to obtain depressed and nondepressed data sets. Some manual screening was implemented to remove “fake” depression microblogs from the data sets, which is clarified in the “Data Preprocessing” subsection. We extracted 80 words as seeds and then built a semantic association graph with the similarities between the seeds and candidate words and utilized the label propagation algorithm (LPA) to automatically mark new words in the graph. The LPA is a good method in such a construction, which has been further explained in the “Related Work” subsection. We then tested the effectiveness of this method and compared it with some baseline approaches. We found that this autoconstruction of a depression-domain lexicon performed the best and had the most stable performance when parameters changed. For further research, this lexicon was used as an input for machine learning algorithms, providing insights into the depressive status of test subjects, so as to improve detection accuracy. According to our research, the detection models with lexicon features outperformed the models without lexicon features by 2% to 9% in terms of evaluation scores.

The main contributions are as follows: (1) We extracted a set of depressive words and constructed our domain lexicon in Chinese, which is a good contribution to web-related depression signal detection, to assist in identifying users who have the potential to experience depression in an early period. We applied an efficient semisupervised automatic construction method in the depression domain. The lexicon was proven to be meaningful in several detecting classification models in our study; (2) We constructed a benchmark depression data set (some of the data were used to construct the lexicon [our main research objective] and the other data were used in the detection test) based on microblogs, which could assist in further depression detection, diagnosis, and analysis. Meanwhile, we released the data set and lexicon together [15] to facilitate future web-related depression diagnosis.

### Related Work About the Traditional Approach for Depression Detection

For decades, there have been many ways to detect depression. Beck [16] created the original Beck Depression Inventory for a quick self-testing measure that can briefly assess recent depression symptoms. Thereafter, Beck et al [17] updated the approach to Beck Depression Inventory II that can assess the severity of self-reported depression symptoms by paper or electronic format. Radloff [18] developed the Center for Epidemiologic Studies Depression Scale, which focuses more on the individual’s emotional experience and less on the physical condition. Some other popular scales are the Zung Self-rating Depression Scale [19] and Hamilton Depression Rating Scale [20].

Since the 21st century, new scales are continuously being improved. Diagnostic and Statistical Manual of Mental Disorders (DSM-IV) is a standard classification of mental disorders used by mental health professionals, which was improved by Hu [21]. In China, the Chinese Classification and Diagnosis of Mental Diseases 3rd edition (CCMD-3) is a standard for diagnosis.

Overall, traditional ways of depression detection have been highly validated and accepted in the real world for decades. However, they mainly rely on the scores of scales or questionnaires, face-to-face interviews, and self-reports, and often require a lot of labor and time [6]. The new trend might be related to big data that are timely, rich, and easily accessible on social networking sites like Facebook, Twitter, and Weibo. These web-based methods can assist in large-scale early detection, and experts can further conduct more precise diagnosis and treatment.

### Related Work About Depression Detection on Social Media

In recent years, with abundant data on social media, some researchers are attempting to detect depression by leveraging web-based data. Park et al [8] explored the use of depressive language from Twitter users and concluded that social networks can provide meaningful data for capturing depressive moods. Choudhury et al [9] were the first to diagnose and predict depression via social media by extracting several features. Hasan et al [10] proposed a new method with the circumplex model to classify Twitter messages as depressed, happy, or other emotions. Resnik et al [11] explored the use of supervised topic models in an analysis of linguistic signals for detecting depression. During such research, word-based features are of great importance on social media [14].

Word-based features could be shown in a lexicon. Tsugawa et al [12] utilized positive and negative sentiment words in a lexicon for recognizing depression. Choudhury et al [9] used semantic orientation pointwise mutual information (SO-PMI) and term frequency-inverse documentation frequency (TF-IDF) to extract a depression lexicon from “mental health” in Yahoo! Answers and set the Wikipedia page on “list of antidepressants” as antidepressant words. Most recently, Guangyao et al [13] employed Word2Vec (W2V) to extract words of antidepressants and depression symptoms from Twitter as a domain-specific lexicon.

Many previous studies achieved a lot with regard to a depression lexicon, which can greatly help in diagnosis; however, most of the words are in English. It is not proper to use the translated version of an English lexicon to detect depression in a Chinese corpus because of cultural differences. In addition, only PMI (mainly about co-occurrence frequency) and W2V (word embeddings) techniques cannot keep up with today’s semantic developments. We can see the feasibility of detecting depression on social media with a lexicon, and more efforts are needed to construct a better Chinese depression-domain lexicon.

### Related Work About Research on Construction of a Domain Lexicon

Many methods have been used to efficiently construct a domain lexicon. Das et al [22] and Krestel and Siersdorfer [23] used SO-PMI as a useful tool for emotion lexicon construction. Yu and Dredze [24], Tixier et al [25], and Zhengyu [26] leveraged and improved the W2V method to construct a domain lexicon. Chao et al [27] proposed a semisupervised sentiment orientation classification algorithm based on W2V (SO-W2V) and obtained a lexicon in different areas efficiently. The PMI method focuses on the co-occurrence frequency between words but ignores the context. However, W2V considers context with word embeddings but in a relatively simple way compared with the LPA shown below.

The LPA, which was first proposed by Zhu and Ghahramaniy [28], plays an important role in lexicon autoconstruction with semisupervised methods. Researchers [29,30] used the LPA starting with several labeled seed words to expand a lexicon for polarity classification. Tai and Kao [31] built a framework to automatically generate a lexicon by combining PMI and the LPA. Hamilton et al [32] applied a label propagation framework with domain-specific word embeddings to construct accurate domain-specific lexicons. A new method combining W2V and LPA was adopted by Giulianelli [33] and Pu et al [34], and it performed much better than previous methods. In this way, the relationships between words and specific domain contexts are considered.

### Data Collection

In order to build a depression-domain lexicon for further detection via social media, we constructed two data sets of users with depression and without depression based on data from Weibo microblogs, which is very popular in China. Weibo has 462 million monthly active users according to a report in 2018 [35], and it is the most popular social media website in China. Equivalent with Twitter, people are getting used to sharing their ideas and moods on Weibo.

In light of the fact that depression is a long-standing illness, the text of users should not be collected from only one microblog. Thus, our data sets contained all Weibo microblogs within a year published by the same users. In addition, personal profile information like comments, number of follows, and number of followers was also included.

#### Depressed Data Set D1

Based on Weibo microblogs from January 2017 to April 2018, we used the keywords “I’m diagnosed with depression” [13,36,37] to construct a depressed data set *D*1. In this way, we finally identified 965 users with depression and 58,265 microblogs (Table 1).

**Table 1:** Details of the collected data sets from Weibo microblogs.

Data set	Users	Total posts	Mean	Standard deviation	Skewness	Kurtosis	Time span
Depressed data set *D*1	965	58,265	60.374	31.327	−0.451	1.788	January 2017-April 2018 (16 months)
Nondepressed data set *D*2	903	52,787	63.697	30.086	−0.615	2.066	January 2017-April 2018 (16 months)

#### Nondepressed Data Set D2

If a user never posted any text with a depression-related word like “depress,” the user was labeled as nondepressed. In this way, we constructed a nondepressed data set *D*2. To match *D*1, we selected a similar number of microblogs (one user without depression can have up to 100 posts) under the same time span. In this way, we identified 903 users without depression and 52,787 microblogs (Table 1).

### Data Preprocessing

Before the experiment, we found that there were some unrelated microblogs, irregular words, and emoji in our data sets. These noisy texts can affect the accuracy of our model, so we adopted the following preprocessing procedures: (1) *Emoji processing*. Emoji and emoticons are common in social media. However, they can cause some unexpected troubles like encoding problems during algorithm running and text analysis, so we removed emoji. We will take them into account separately in further research; (2) *Unrelated* *microblog processing*. In addition to depression-domain microblogs that we focused on mostly, many users posted plenty of daily microblogs, including red packets snatching, game sharing, advertisements, etc. In addition, some “fake” depression microblogs like depression scientific articles and content talking about friends with depression, instead of users, are also useless and can be misleading. By manual screening, we obtained a list of unrelated keywords in daily microblogs and “fake” microblogs, and then, we removed them all with regular expression; (3) *Irregular words preprocessing.* New words keep appearing, and language habits are quite different on the internet. These cause trouble during text analysis. Therefore, we added a general dictionary of internet words.

## Methods

### Construction Overview

Domain adaptability is always a difficult problem in natural language processing. Therefore, a domain-based lexicon can help us perform analysis in a more accurate and deeper way. For example, “excitement,” “life,” and “forever” are common words in our daily life, but they can be abnormal signals of a patient with depression. Thus, through our study, we will try our best to determine which words used on the internet indicate depression and which do not indicate depression.

There are many ways to construct a domain-based lexicon according to a survey [38], which can mainly be divided into knowledge base, corpus base, and these two combined. Knowledge base includes traditional methods, such as word relation extension [39] and annotation extension [40]. Corpus base refers to conjunction syntax [41] and word co-occurrence [42]. In fact, a survey [38] showed that the class of methods adopted more widely is the automatic method combining existing knowledge and corpus base. In this view, approaches involving semisupervised construction on relationship graphs like the LPA, bootstrapping, and word embedding are popular and effective methods mentioned in the subsection Related Work About Research on Construction of a Domain Lexicon.

**Figure 1:**
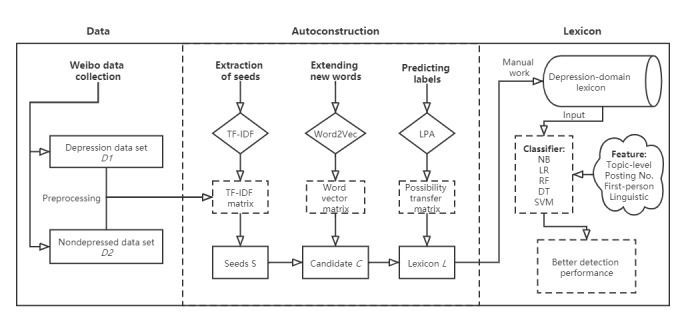
An illustration of the framework. DT: decision tree; LR: logistic regression; NB: naive Bayes; RF: random forest; SVM: support vector machine; TF-IDF: term frequency-inverse documentation frequency.

Inspired by Hamilton [20], Giulianelli [33], and Pu et al [34], we applied automatic construction, a semisupervised method that combines W2V and LPA on a lexical semantic relationship graph, to obtain a depression-domain lexicon containing depressed feature words. Our construction can be divided into the following four steps: (1) *Extraction of seed words*. Extract a few words that are most important and valuable in the domain; (2) *Extending new words*. Use the W2V model to learn word vectors on the corpus and then extend with similarity between seeds and new words; (3) *Setting labels for the new words*. If the cosine similarity of a word and a seed is greater than the threshold, an edge will be formed, and the weight of the edge is the similarity. Through such iteration, a graph is obtained. After that, the LPA is used on the semantic graph to obtain the labels of all candidate words; (4) *Obtaining the domain-based lexicon*. Finally, by simple manual arrangement, the depression-domain lexicon is formed. We then used it as an input for detection models and found that the models performed much better than before. The method is described as a detailed framework in Figure 1.

### Extraction of Seed Words

Seed words are those that can be representative of a specific domain. In order to extract the key seed words in the depressed and nondepressed data sets, we leveraged the TF-IDF algorithm, which is a widely used feature extraction algorithm in natural language processing. Salton and Yu [43] first proposed the TF-IDF algorithm, and Salton et al [44] demonstrated its validity in information retrieval. Term frequency (TF) refers to the number of times a term or word occurs in a document, and inverse document frequency (IDF) is related to the frequency of a term appearing in all documents, which measures specificity of the term over the entire corpus.

TF and IDF can be formulated as follows:


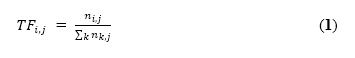



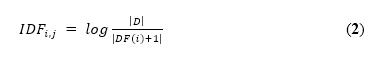


where *n_i,j_* is the word *i* in document *j*, *k* is the number of words in *j*, *N* is the number of documents containing word *i*, *D* is the size of the documents, and *DF(i)* is the number of documents in which the word *i* occurs at least once. Additionally, *tfidf* can be formulated as follows:


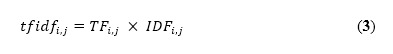


Intuitively, this calculation of TF-IDF will show us how important and special a given word is in our depression domain. Words with a higher *tfidf* value tend to have a greater relevance in a document. In our research, we regarded the data sets *D*1 and *D*2 as two corpora and every microblog as a document. We then extracted words with the highest TF-IDF values in our corpora.

### Extending New Words With W2V

Now that we had the seeds *S*, we could leverage the word embedding model to extend new words. Word embeddings, which help map the vocabulary to vectors, are popular tools for natural language processing. We adopted W2V, an efficient algorithm for learning embeddings using a neural language model, to generate the vectors. W2V is an open source model by Mikolov et al [45] at Google, and its main idea is to use deep learning technology on a specific corpus and then to map each word into a multidimensional real vector space, where the distance between words that have a higher semantic similarity is small.

In this paper, cosine similarity was used to calculate the similarity between words. When a word whose similarity with the seed words in the training corpus was greater than the given threshold, we extracted it as a new word and added it as a candidate word to the candidate word set *C*. If S*_i_* and C*_j_* represent the vectors of a seed word and candidate word, respectively, the similarity between them can be formulated by *SIM*(*Si*, *Cj*) as follows:


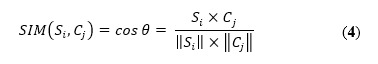


### Setting Labels With Label Propagation

The LPA is a common semisupervised approach on a graph [28]. It has been applied to many fields, such as community detection [46], personal social relation extraction [47], and dictionary construction. Using a graph model to construct a lexicon can capture the global relations among all words, overcome the dependence on seeds, and provide a better result in the case of limited labeled data.

The LPA builds a graph based on the similarity between nodes, which are the words in our study. After the initialization of the graph, the nodes in the graph can be divided into labeled nodes and unknown nodes. The basic idea of LPA is to predict the label of unknown nodes based on information from labeled ones, and labels are propagated mainly by the weight on the edge between the nodes. In the process of label propagation, unknown nodes can update their own labels through the information of adjacent known labels. If the similarity of the adjacent node is large, the influence of its label will be large.

**Figure 2:**
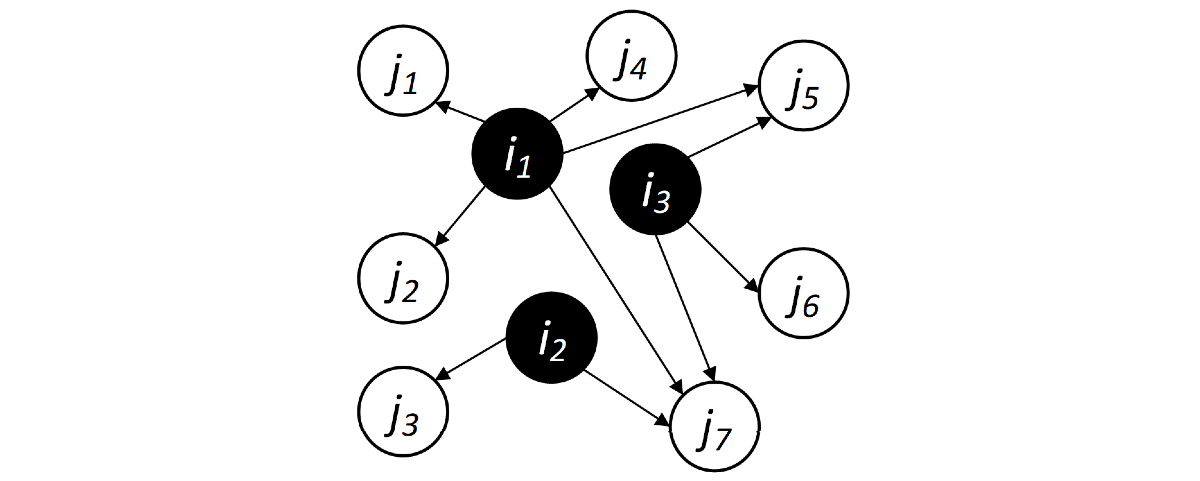
A simple structure of a semantic graph. i: seed word; j: candidate word.

In our algorithm, the seeds *S* are taken as the labeled nodes and the extended candidate words *C* are taken as the unknown nodes. The semantic graph is constructed as follows: If the seed word *i* is extended by W2V to get a new word *j*, there is an edge between *i* and *j*, and the weight of the edge is the similarity of the two words. Thus, all of the seed words and candidate words will form a semantic graph as shown in Figure 2.

Assuming that there are *n* nodes in total, then an *n*-dimensional transition probability matrix can be constructed. Let *SIM*(*w_i_*,*w_j_*) represent the similarity between *w_i_* and *w_j_*, which is calculated by cosine similarity. *T*[*i*][*j*]represents the similarity transfer probability from word *i* to word *j*, which is calculated as follows:


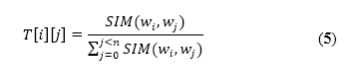


If there are 10 nodes in the graph, in which *i_1_* and *i_2_* are depression seed words with the label “−1,” *i_3_*is a nondepression seed word with the label “+1,” and the labels of the rest of the candidate words are unknown (given an initial value of 0), the initial labels of all nodes can be represented by the vector *V* as follows:


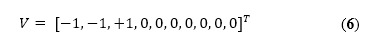


The label of each unknown candidate word is obtained by iteratively applying the transition matrix on the initial labels of the words. The calculation method is as follows:


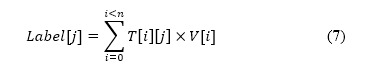


where *Label* represents the label possibility of node *j* after the iteration, *T*[*i*][*j*] represents the transfer probability in the similarity matrix of node *i* to node *j*, and *V*[*i*] represents the initial *Label* of node *i* before the iteration.

In each iteration, the labels of the seeds should remain the same. When the labels of all words in the graph no longer change after continuous iteration, the iteration is over. At the end of the iteration process, the final candidate words are those words whose absolute value of label probability is greater than a certain threshold. In this way, we obtained a well-labeled domain lexicon. The previous algorithms can be concluded as the steps in Textbox 1.

**Algorithms of the procedure.**

***Input:*** Corpus of data set (Corpus=*D*1∪*D*2), seeds *S*, and the threshold *T_c_* for candidate words *C*.

***Output:*** One depression-domain lexicon *L* with depressive words *L_d_* and nondepressive words *L_n_*.

***Procedure:***

1) Initialize the lexicon and candidate words. *C*=∅, *L_d_*=∅, *L_n_*=∅.

2) Preprocess the corpus and learn the word embedding with Word2Vec.

3) For every seed, *S_i_*∈*S*:

For a word *W_j_* in *Corpus*, if *SIM*(*S_i_*,*W_j_*) ≥*T_c_*, then *C*=*C*∪*S*_i_∪*W*_j_. Record the similarity calculated by equation (4).

4) After obtaining all the extended candidate words *C* and the similarity matrix between words through step 3), the transition probability matrix of similarity in *C* can be constructed according to equation (5). Then, the semantic relationship graph is obtained.

5) In the whole graph, *Label* of unknown words is calculated according to formula (7) given the initial label *V*.

6) Reset the labels of the seeds in *Label* to its initial value. Then, let *V*=*Label*.

7) Repeat steps 5) and 6) until the labels of *C* in the graph do not change anymore.

8) Obtain the final *Label*. For *C_i_*∈*C*, if *Label_Ci_* <0 and |*Label_Ci_*| >0.5, then *L_d_*=*L_d_*∪*C_i_*; For *C_j_*∈*C*, if *Label_Cj_* >0 and |*Label_Cj_*| >0.5, then *L_n_*=*L_n_*∪*C_j_*.

9) Combine *L_d_* and *L_n_* and finally obtain the depression-domain lexicon *L* after manual work.

## Results

### Experiment Setup

We employed our data set to construct a depression-domain lexicon. We needed two types of microblogs combining users with depression or those without depression to extract domain seed words and to finish the automatic construction with the LPA. Our original data crawled from Weibo had some noise, especially in *D*1, so manual preprocessing (detailed description in the “Data Preprocessing” subsection) was necessary to clean the data into *D*1 and *D*2.

After our lexicon was automatically built, we labeled it depressed or nondepressed for further evaluation. Three volunteers, who had carefully read the depressed microblogs and research articles, were invited to perform the lexicon labeling job [48]. Thus, every word in the lexicon was labeled three times. If there was a labeling disagreement, voting was adopted to obtain the ground truth.

### Word Segmentation

Chinese word segmentation has a great influence in lexicon construction, especially when it comes to Weibo microblogs and the depression domain. In order to segment Chinese words properly in Weibo text, we used the following three steps to segment words as accurately as possible: (1) domain dictionary; (2) large word embedding; and (3) incorrect word removal.

#### Domain Dictionary

When coping with mental disease, especially depression, over the internet, some depression-domain words like paroxetine (“帕罗西汀”), which is a common antidepressant, and self-rating scale (“自评量表”), which is a tool for individuals to measure depression, were difficult to recognize. Other words like MLGB (“马勒戈壁”), which means damn it, and Yali (“鸭梨”), which means pressure, were network vocabularies that could be confusing for the computer. Domain-specific segmentation should combine a domain dictionary [49]; however, there is no depression dictionary in public resources. To solve the segmentation problem, we downloaded “Dictionary of Psychology” and “Dictionary of Neuropsychiatry” from the CNKI Tool library [50] (there is no depression lexicon yet, so we chose the dictionary of psychology and psychiatry; CNKI is one of the largest Chinese knowledge discovery web-based platforms), downloaded “Weibo Dictionary” from BosonNLP [51] (a dictionary automatically constructed from millions of annotation data points from microblogs, forums, and other data sources), and used a manually collected antidepressant dictionary [26] (words like amitriptyline and paroxetine in our data sets were replaced with antidepressant as a data reduction method) from web-based pharmacies and science articles. The work of Chinese domain word segmentation was inspired by Fang [26] and Cheng [49]. The final domain dictionary contained 122,594 words after eliminating duplicate words. We then used jieba (built to be the best Python Chinese word segmentation module) [52] as our segmentation module, which adopted the unigram model and hidden Markov model.

#### Large Word Embedding

A richer corpus is associated with more precise word embedding. Instead of using our collected data, which were relatively rare, we leveraged the W2V models by Shen et al [53], which are trained on 5 million Weibo microblogs and 223 million Chinese Wiki tokens, for word embeddings.

#### Incorrect Word Removal

We planned to remove incorrect words from our lexicon. Actually, after evaluation, we found that the error rate was less than 2% to 3%. Among 2385 words in our depression-domain lexicon, there were 64 errors.

### Evaluation Metrics

During our experiments, we constructed the depression-domain lexicon with an automatic method, compared our method with some baseline approaches, and analyzed key parameters like number of seeds and threshold in the model.

For the evaluation metrics, we employed precision, recall, and F1 measure (F1) in equations (8), (9) and (10), respectively, to evaluate the performance of our model and the baseline approaches. We used area under the curve (AUC) to evaluate the model of unbalanced data. In terms of the number of words in the lexicon, we also compared the numbers under different circumstances. The equations are as follows:


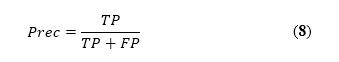



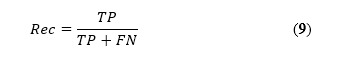



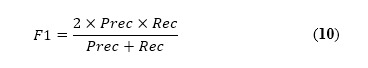


where *TP* represents true positive, which means depressed words are correctly detected as depressed; *FN* is false negative, which means depressed words are incorrectly determined as nondepressed; and *FP* is false positive, which means that nondepressed words are incorrectly detected as depressed.

Figure 1 provides an entire picture of the experiment.

### Seed Words

Before construction, we used the TF-IDF to extract the seed words and obtained a list of the top 2000 words. The samples of the TF-IDF of *D*1 are shown in Table 2.

By artificially screening the list, we could obtain some seed words. Moreover, we added a few general sentiment words with high levels to our seed words and finally obtained a set of seed words of 40 depressive seeds and 40 nondepressive seeds. From parameter sensitivity analysis, we noted that 80 seeds in total will lead to a sufficiently large lexicon with high accuracy. The samples of the 80 seeds are shown in Table 3.

**Table 2:** TF-IDF values of depressed D1 samples.

Depressed *D*1	TF-IDF^a^ value
Myself (自己)	0.041383
Really (真的)	0.032475
Depression (抑郁症)	0.024328
Hope (希望)	0.013336
Life (生活)	0.012043
Forever (永远)	0.006965
Pain (痛苦)	0.006871
Sad (难过)	0.006756
Live (活着)	0.006583
Mood (心情)	0.006386
Night (晚上)	0.006347
Always (总是)	0.005984
Hate (讨厌)	0.005475
Exhausted (好累)	0.005469
Fear (害怕)	0.005030
Lonely (孤独)	0.004413
Idiot (傻逼)	0.004380
Emotion (感情)	0.004031
Insomnia (失眠)	0.003950
Sorry (对不起)	0.003867
Despair (绝望)	0.003410
Antidepressant (抗抑郁药)	0.002305

^a^TF-IDF: term frequency-inverse documentation frequency.

**Table 3:** Summary of the seeds.

Category	Seeds *S*
Nondepressive (40 words)	Stability, comfort, happy, happiness, successful, confidence, sunshine, struggle, positive, brave, enjoy, peace, enthusiasm, healthy, satisfied, active, grow up, pride, good, admire, strong, perfect, praise, precious, progress, congratulate, love, welcome, kindness, robust, earnest, agree, support, award, advantage, good deal, develop, warm, bright colored, and understand (稳定, 舒服, 高兴, 幸福, 顺利, 自信, 阳光, 奋斗, 积极, 勇敢, 享受, 平安, 热情, 健康, 满意, 活力, 成长, 骄傲, 优秀, 敬佩, 完美, 称赞, 强大, 珍贵, 进步, 庆贺, 关爱, 欢迎, 强壮, 善良, 认真, 同意, 支持, 奖励, 优势, 划算, 发展, 温暖, 鲜艳, 明白)
Depressive (40 words)	Depression, collapse, stress, suicide, apastia, anxious, sad, tired, death, lonely, insomnia, bad, desperate, give up, low, leave, fear, danger, close, sensitive, lost, shadow, destroy, suspect, crash, dark, helpless, guilt, negative, frustration, nervous, melancholy, rubbish, jump, forget, goodbye, cut wrist, edge, haze, and antidepressant (抑郁, 崩溃, 压力, 自杀, 绝食, 焦躁, 伤心, 疲惫, 死亡, 孤独, 失眠, 难受, 绝望, 放弃, 卑微, 离开, 恐惧, 危险, 封闭, 敏感, 茫然, 阴影, 摧毁, 怀疑, 崩塌, 黑暗, 无助, 愧疚, 负面, 沮丧, 紧张, 忧郁, 废物, 跳楼, 遗忘, 再见, 割腕, 边缘, 阴霾, 抗抑郁药)

### Model Evaluation

In order to verify the effectiveness of the lexicon autoconstruction method applied in this paper, we selected the following methods as baseline approaches: (1) *W2V* [24-26]. A common method of constructing a lexicon based on W2V, which is used to learn word embedding vectors on a corpus. The semantic similarity between words and seed words in the corpus is then iteratively calculated. If the similarity is greater than a certain threshold, the new word is extended and has the same label as the seed word; (2) *SO-W2V* [27]. It is a semisupervised sentiment orientation classification algorithm based on a word vector. The basic idea is that through comparison with all positive and negative seed words, an accurate orientation of the extended word will be obtained. It has versatility in different areas for a Chinese corpus; (3) *SO-PMI* [9,22,23]. It calculates the probability of the occurrence of both seed words and expanded words in the text. A higher probability is associated with a closer correlation; (4) *W2V-LPA*. It is our method, which considers both the word relationship and specific domain context.

To obtain a fair comparison, we set the same parameters for all methods where *T_c_* was 0.5 and the size of seeds *S* was 80. For W2V tools, we used the gensim package [54].

**Figure 3:**
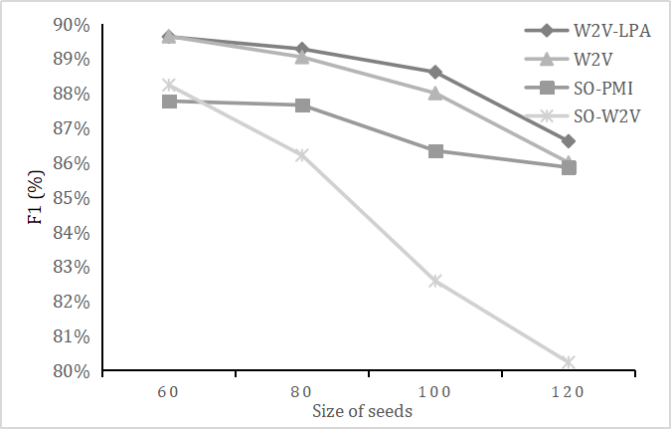
F1 of methods when the seed size changed. LPA: label propagation algorithm; SO-PMI: semantic orientation pointwise mutual information; SO-W2V: semantic orientation from Word2Vec; W2V: Word2Vec.

From Table 4 and Figure 3, we can see the evaluation results. It is obvious that the W2V-LPA and W2V methods performed much better than the SO-W2V and SO-PMI methods. Moreover, when the size of seeds increased from 60 to 120, our method was able to maintain a more stable and precise performance, which was almost 1% to 6% higher than others (Figure 3), whereas the value for SO-W2V declined quickly when the size of seeds became larger. Overall, SO-W2V takes all the other seeds into account, but too many seeds combined will introduce too much noise to some extent, as not all seeds are related to an extended word. W2V is a simple and general method, which only considers the label of the first seed when extending new words. Additionally, SO-PMI mainly takes word co-occurrence frequency into account. What W2V-LPA did better is that it only predicted labels through the semantic graph of related and similar words, and thus, the semantic context and word relation were both considered. Therefore, we can say that W2V-LPA is a much better and more stable method for the autoconstruction of a domain lexicon.

**Table 4:** Performance of lexicon construction methods.

Construction method	Precision	Recall	F1	Size of the lexicon
W2V-LPA^a^	0.880	0.906	0.893	2321
W2V^b^	0.878	0.903	0.890	2321
SO-PMI^c^	0.879	0.877	0.877	2024
SO-W2V^d^	0.854	0.877	0.862	2321

^a^W2V-LPA: label propagation algorithm-Word2Vec.

^b^W2V: Word2Vec.

^c^SO-PMI: semantic orientation pointwise mutual information.

^d^SO-W2V: semantic orientation Word2Vec.

### Parameter Sensitivity Analysis

**Figure 4:**
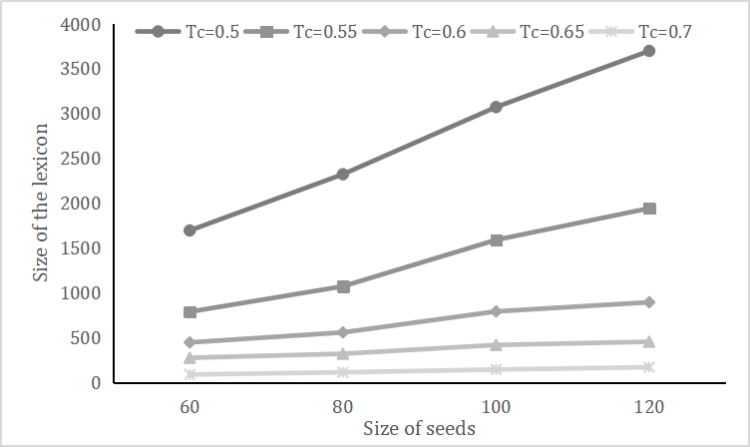
Size of the lexicon when the size of seeds and threshold for candidate words changed.

Throughout our experiment, the size of seeds *S* and the extension threshold *T_c_* were two important parameters. More seeds or a lower threshold will lead to a lexicon with more words but lower accuracy, whereas fewer seeds and a high threshold will ensure more precision but a poor lexicon. We balanced the trade-offs, as we wanted to obtain a relatively accurate and abundant lexicon that would be helpful for further depression diagnosis. Figure 4 presents the size of the lexicon when the size of seeds and threshold for candidate words changed.

First, we fixed *T_c_* at 0.7 and then varied the size of seeds from 60 to 120. If we have less than 60 seeds, the entire lexicon will be so small that almost nothing will remain but seed words. A size larger than 60 will not change the outcome, so 0.7 might be a very high-level threshold. From Table 5, we can see that larger sizes of seeds like 100 and 120 partially jeopardized the performance, and W2V-LPA performed nearly the same when the sizes were 60 and 80.

We then fixed the size of seeds at 80 with varying *T_c_* from 0.7 to 0.5. With a higher threshold, the performance was relatively excellent, whereas the size of the lexicon started to fail at around 1000 when *T_c_* was 0.55. We believe a lexicon with 2000 words and a *T_c_* of 0.5 might have good balance.

Overall, it is pleasing that our W2V-LPA method performed quite smoothly and steadily even when the parameters were changed, so we believe that a high-quality lexicon can be constructed. It is difficult to find an optimal solution, and given *D*1 and *D*2, we will adopt a size of seeds of 80 and a threshold *T_c_* of 0.5 as a relatively proper approach.

**Table 5:** Performance of the W2V-LPA method when S and *T_c_* were changed.

*S* ^a^	*T_c_* ^b^	Precision	Recall	F1	Size of the lexicon
60	0.5	0.882	0.911	0.896	1694
60	0.55	0.910	0.935	0.922	788
60	0.6	0.926	0.944	0.935	446
60	0.65	0.951	0.963	0.954	275
60	0.7	0.804	0.897	0.848	89
80	0.5	0.880	0.906	0.893	2321
80	0.55	0.916	0.937	0.926	1072
80	0.6	0.934	0.948	0.941	558
80	0.65	0.954	0.963	0.958	320
80	0.7	0.918	0.909	0.892	113
100	0.5	0.874	0.899	0.886	3070
100	0.55	0.906	0.924	0.915	1589
100	0.6	0.927	0.937	0.931	792
100	0.65	0.953	0.959	0.955	418
100	0.7	0.937	0.932	0.925	144
120	0.5	0.855	0.879	0.866	3696
120	0.55	0.889	0.904	0.896	1942
120	0.6	0.924	0.933	0.928	894
120	0.65	0.952	0.958	0.954	454
120	0.7	0.944	0.940	0.934	170

^a^*S*: size of the seeds.

^b^*T_c_*: threshold for candidate words.

### Detection Performance

After construction of the depression-domain lexicon, we could apply it to actual depression detection in a new Weibo microblog data set to find out if our work would help existing detection models perform better. The detection process included data collection, feature selection, and classification methods.

#### Data Collection

In addition to our data set used for lexicon construction, we collected 745 users who were depressed and 10,118 users who were not depressed with their 1-year tweets as a new data set. Data details are shown in Table 6.

**Table 6:** Details of the data set for depression detection.

Data set	Users	Total posts	Mean	Standard deviation	Skewness	Kurtosis	Time span
Depressed data set *D*3	745	179,600	240.44	486.28	6.21	56.32	January 2018-June 2019 (18 months)
Nondepressed data set *D*4	10,118	3,150,000	310.93	327.72	3.50	48.52	January 2018-June 2019 (18 months)

#### Feature Selection

Features like topic-level keywords, posting behaviors, number of tweets, first-person words, and linguistic style are meaningful in detecting depression on the internet [11,13]. We also set our depression-domain lexicon as one feature to see whether it would really contribute a lot after inclusion in the detection model. The features were as follows: (1) *Topic-level keywords.* We selected 30 topic-level keywords with the TF-IDF; (2) *Posting behaviors.* For each user, average length of tweets and total posting numbers were collected to represent web-related posting behaviors; (3) *First-person words.* According to linguistic inquiry and word count [55], we counted the number of first-person pronouns like I, we, us, etc; (4) *Linguistic style (200 dimensions).* To approximately analyze linguistic style, we calculated the average vectors of every user with Word2Vec [56]. Finally, we constructed the depression-domain lexicon by the previously mentioned process.

#### Classification Methods

We chose naive Bayes (NB), decision tree, logistic regression (LR), random forest, and support vector machine [5,37] as classification methods to detect users with depression. From model performance, we obtained a quick picture about the importance of our lexicon. When the depression-domain lexicon is selected as one feature, the method has the tag L. For example, L-NB is a classification algorithm that has the feature of the depression-domain lexicon, whereas NB does not have this feature. After including the depression-domain lexicon in the models, we clearly found that each detection performance improved when compared with before inclusion of the lexicon (Table 7). The performance of lexicon methods surpassed that of corresponding methods without the lexicon by 2% to 9%, which justifies the important role of our lexicon in depression detection.

**Figure 5:**
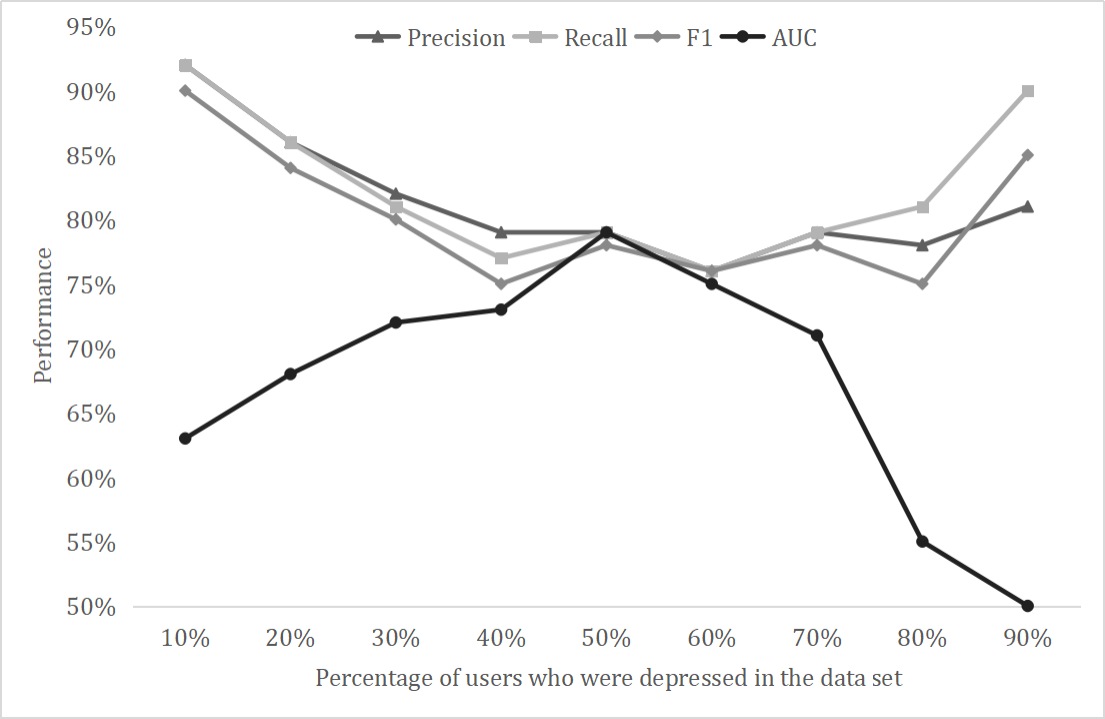
Scales of users who were depressed. AUC: area under the curve.

The model was based on a data set with 50% users who were depressed and 50% users who were not depressed. When we varied the scale of depressed users, the data set became imbalanced and the AUC was more important to test the performance. Figure 5 illustrates the trend of detecting performance when setting different proportions of users who were depressed in the L-LR method. This method achieved an outstanding performance when the proportion of users with depression was 50%. However, the AUC dropped sharply when the data set was imbalanced.

In the real word, people with depression make up less than 10% of the population, and we will determine how to properly detect depression with imbalanced data in a further study.

**Table 7:** Detection model performance with the depression-domain lexicon.

Detection model	Precision	Recall	F1	Accuracy
NB^a^	67%	67%	67%	67%
L^b^-NB	74%	73%	73%	73%
LR^c^	76%	76%	75%	76%
L-LR	77%	77%	77%	77%
RF^d^	68%	68%	68%	68%
L-RF	77%	77%	76%	77%
SVM^e^	65%	65%	65%	65%
L-SVM	74%	72%	72%	72%
DT^f^	67%	67%	67%	67%
L-DT	69%	69%	69%	69%

^a^NB: naive Bayes.

^b^L: depression-domain lexicon as a feature.

^c^LR: logistic regression.

^d^RF: random forest.

^e^SVM: support vector machine.

^f^DT: decision tree.

## Discussion

Diagnosis of users with potential depression via social media has attracted increasing attention because it is a more cost-effective and active approach dealing with massive valuable data than traditional diagnosis. In previous studies, most of the achievements about a lexicon involved an English corpus. Instead of translating an English lexicon, this paper aimed to apply an automatic construction method for a Chinese depression-domain lexicon based on the LPA. With Word2Vec and a semantic relationship graph, the LPA was used to predict the label of candidate words in the graph, and finally, our lexicon was constructed. Experiment results showed that our method was superior to baseline construction methods and had good performance and robustness. In addition, when our lexicon was included as an input for the detection models, their performance became more accurate and effective when compared with the models without the depression-domain lexicon.

In the next step, experiments are expected to be carried out on a larger depression corpus, and more linguistic knowledge like conjunction will be incorporated into our method to enlarge the range of the depression-domain lexicon. Meanwhile, more complex construction methods like deep neural networks and hierarchical topic models will be adopted in further research. We expect that our lexicon will act as a useful feature in depression detection and will be able to provide more insights for depression diagnosis in terms of advanced depression detection among patients.

## Abbreviations

DT: decision tree
LPA: label propagation algorithm
LR: logistic regression
NB: naive Bayes
RF: random forest
SO-PMI: semantic orientation pointwise mutual information
SO-W2V: semantic orientation from Word2Vec
SVM: support vector machine
TF-IDF: term frequency-inverse documentation frequency
W2V: Word2Vec
